# The impact of temporal lobe epilepsy surgery on picture naming and its relationship to network metric change

**DOI:** 10.1016/j.nicl.2023.103444

**Published:** 2023-05-27

**Authors:** Lawrence Peter Binding, Peter Neal Taylor, Aidan G. O'Keeffe, Davide Giampiccolo, Marine Fleury, Fenglai Xiao, Lorenzo Caciagli, Jane de Tisi, Gavin P. Winston, Anna Miserocchi, Andrew McEvoy, John S. Duncan, Sjoerd B. Vos

**Affiliations:** a Centre for Medical Image Computing, Department of Computer Science, UCL, London, United Kingdom; b Department of Clinical and Experimental Epilepsy, UCL Queen Square Institute of Neurology, London, United Kingdom; c CNNP lab, Interdisciplinary Computing and Complex BioSystems Group, School of Computing Science, Newcastle University, United Kingdom; d School of Mathematical Sciences, University of Nottingham, United Kingdom; e Institute of Epidemiology and Healthcare, UCL, London WC1E 6BT, United Kingdom; f Victor Horsley Department of Neurosurgery, National Hospital for Neurology and Neurosurgery, Queen Square, London, United Kingdom; g Department of Neurosurgery, Institute of Neurosciences, Cleveland Clinic London, United Kingdom; h MRI Unit, Chalfont Centre for Epilepsy, Chalfont St Peter, United Kingdom; i Department of Medicine, Division of Neurology, Queens University, Kingston, Canada; j Department of Clinical and Experimental Epilepsy, UCL Queen Square Institute of Neurology, London, United Kingdom; k Neuroradiological Academic Unit, UCL Queen Square Institute of Neurology, University College London, London, United Kingdom; l Centre for Microscopy, Characterisation, and Analysis, The University of Western Australia, Nedlands, Australia

**Keywords:** White Matter, Anterior Temporal Lobe Resection, Picture Naming Decline, Graph Theory, Machine Learning

## Abstract

**Background:**

Anterior temporal lobe resection (ATLR) is a successful treatment for medically-refractory temporal lobe epilepsy (TLE). In the language-dominant hemisphere, 30%- 50% of individuals experience a naming decline which can impact upon daily life. Measures of structural networks are associated with language performance pre-operatively. It is unclear if analysis of network measures may predict post-operative decline.

**Methods:**

White matter fibre tractography was performed on preoperative diffusion MRI of 44 left lateralised and left resection individuals with TLE to reconstruct the preoperative structural network. Resection masks, drawn on co-registered pre- and post-operative T1-weighted MRI scans, were used as exclusion regions on pre-operative tractography to estimate the post-operative network. Changes in graph theory metrics, cortical strength, betweenness centrality, and clustering coefficient were generated by comparing the estimated pre- and post-operative networks. These were thresholded based on the presence of the connection in each patient, ranging from 75% to 100% in steps of 5%. The average graph theory metric across thresholds was taken.

We incorporated leave-one-out cross-validation with smoothly clipped absolute deviation (SCAD) least absolute shrinkage and selection operator (LASSO) feature selection and a support vector classifier to assess graph theory metrics on picture naming decline. Picture naming was assessed via the Graded Naming Test preoperatively and at 3 and 12 months post-operatively and the outcome was classified using the reliable change index (RCI) to identify clinically significant decline. The best feature combination and model was selected using the area under the curve (AUC). The sensitivity, specificity and F1-score were also reported. Permutation testing was performed to assess the machine learning model and selected regions difference significance.

**Results:**

A combination of clinical and graph theory metrics were able to classify outcome of picture naming at 3 months with an AUC of 0.84. At 12 months, change in strength to cortical regions was best able to correctly classify outcome with an AUC of 0.86. Longitudinal analysis revealed that betweenness centrality was the best metric to identify patients who declined at 3 months, who will then continue to experience decline from 3 to 12 months. Both models were significantly higher AUC values than a random classifier.

**Conclusion:**

Our results suggest that inferred changes of network integrity were able to correctly classify picture naming decline after ATLR. These measures may be used to prospectively to identify patients who are at risk of picture naming decline after surgery and could potentially be utilised to assist tailoring the resection in order to prevent this decline.

## Highlights

• Picture naming outcome can be identified via the change in graph theory metrics.
• Combined clinical and graph metrics accurately classifies 3-month decline.
• Change in strength to cortical regions is the best classifier of 12-month decline.
• Outcome across timepoints is best identified by change in betweenness centrality.
• Multiple cognitive domain dysfunction likely underlies picture naming decline.

## 1 Introduction

Language is impaired in up to 50% of patients with temporal lobe epilepsy (TLE) (Reyes et al., 2019). This impairment typically affects naming and verbal fluency (Allone et al., 2017), especially when the epileptogenic zone is within the language dominant hemisphere (Rosazza et al., 2013). Longer duration of disease can be associated with worse cognitive function (Helmstaedter and Kockelmann, 2006). For medically-refractory TLE patients, anterior temporal lobe resection (ATLR) is effective for seizure control. However, individuals undergoing language-dominant resection have a 30–50% risk of significant post-operative decline in language-related functions, particularly naming, which can impact daily life (Sherman et al., 2011, Zhao et al., 2014;17:374.). An ability to minimize the impact of ATLR on language function would be beneficial.

Language function is subserved by synchronized processing in dispersed cortical regions, connected through white matter fibers (Binding et al., 2022). Lateralization of cortical activation in visual and auditory naming functional MRI (fMRI) tasks to the ipsilateral temporal lobe predicts which patients will experience language decline (Trimmel et al., 2019). However, surgically sparing fMRI-activated cortical regions does not avoid a naming impairment in 50% of individuals (Davies et al., 2005). This suggests a possible role of white matter damage in post-surgical language decline.

There is interindividual variation of tract anatomy, functional cortical anatomy, and of tissue resected, including for relatively standardised ATLR, resulting in variability of language outcome. Highlighting this, cortical stimulation in the temporal lobe showed extremely high variability on cortical responses, emphasising a role for plastic reorganisation in individual subjects (Sanai et al., 2008). In those with gliomas, distinct patterns of language reorganisation have been shown according to tumour location (Herbet et al., 2019). Individuals with epilepsy may undergo reorganisation specific to their epileptogenic network, which may be potentially predicted by network analysis.

White matter fibres are anatomically arranged in fibre bundles. Recent work has demonstrated that transection of specific fibre bundles within the temporal lobe contributes to picture naming decline (Binding et al., 2023, Herbet et al., 2016). Fibre bundle analysis, however, is limited by our understanding of white matter fibre anatomy and function. A direct comparison of whole-brain tractography and fibre bundle analysis highlights superiority of the former in predicting postoperative outcome (Kaestner et al., 2020). Previous research utilising graph theory metrics in TLE has demonstrated the centrality metric was able to predict pre-operative picture naming scores to a high degree (Munsell et al., 2019).

There is uncertainty as to whether whole-brain network analysis and graph theory metrics can predict language decline following ATLR. Our aim is to explore whether the estimated change in the cortical region’s network properties after ATLR is able to classify picture naming decline, and ultimately might be used to mitigate such risk. The goal is to understand the disruption of structural connectivity following ATLR that relates to picture naming decline, with the aim of improving future outcomes through enhanced neurosurgical planning.

## 2 Materials and method

### 2.1 Participants

We studied 44 patients with medically refractory left TLE (27 females, mean age: 40 yr) and left lateralised language who underwent ATLR at the National Hospital of Neurology and Neurosurgery, London, United Kingdom between 2010 and 2019. Exclusion criteria were a previous history of neurosurgery, incomplete data, or non-dominant, right, or bilateral language lateralisation. All patients had a pre-operative: T1-weighted structural MRI; dMRI; task-based language fMRI, and a post-operative T1-weighted MRI (obtained between 3- and 12-months post-operatively).

All patients had a typical ATLR, removing the anterior temporal lobe and the majority of the amygdala and hippocampus. Pathology comprised hippocampal sclerosis (HS; N = 27), cavernoma (CAV; N = 2), dysembryoplastic neuroepithelial tumour (DNT; N = 5), dual pathology (N = 6), and other (N = 4).

### 2.2 Standard protocol Approvals, Registrations, and patient consents

This project was approved by London – Bloomsbury Research Ethics Committee (REC reference: 20/LO/0149; CAG number: 20/CAG/0013). Patient data were pseudo-anonymised. This project did not carry any risk to participants and was retrospectively conducted on clinically acquired data.

### 2.3 Neuropsychology

Language was assessed via the McKenna Graded Naming Test which is a visual confrontation naming assessment (referred to as picture naming) (Warrington, 1997). This was performed pre-operatively and 3 and 12 months post-operatively. There were seven patients missing data from the 12 months follow-up. These patients were removed from the 12-month analysis. Change in neuropsychological performance was assessed using the reliable change index (RCI) which was dichotomized to create a binary variable that measured significant decline vs. no decline. An RCI-decline of ≥ 4 was considered a clinically significant decline as per previous research (Trimmel et al., 2019). There were 17/44 (38.6%) and 11/37 (29.7%) patients who had clinically significant picture naming decline at 3 and 12 months, respectively.

### 2.4 Clinical features

Recent evidence suggests that side of surgery, age of onset, and preoperative naming scores are able to predict naming decline with high accuracy (Busch et al., 2018). While we already divided patients based on side of surgery, we extracted the fMRI LI, age at surgery, age of onset and preoperative naming scores for inclusion in our analysis. Table 1 includes the mean and standard deviation for the clinical variables included split across patients who did and did not undergo clinically significant decline (as determined via the RCI). (see Table 2.)

**Table 1:** Descriptive statistics of clinical characteristics. Descriptive statistics are split between those with and without clinically significant decline which is determined by the reliable change index,

	3 Months Decline: Mean (STD)	3 Month No Decline: Mean (STD)	12 Months Decline: Mean (STD)	12 Months No Decline: Mean (STD)
Age of Onset	13.07(9.12)	20.17(14.82)	23.86(17.81)	15.6(10.98)
Preoperative naming score	14.18(6.15)	15.46(5.67)	18.55(5.77)	13.62(5.19)
fMRI LI	0.8(0.13)	0.71(0.2)	0.65(0.26)	0.78(0.14)
Age	40.45(9.09)	40(12.12)	47.91(12.1)	36.85(9.15)

Abbreviations: fMRI LI: functional magnetic resonance imaging lateralisation index; STD: standard deviation.

**Table 2:** Classification capability of clinical and graph theory metrics to picture naming decline.

Timepoint	3 Months		12 Months		Longitudinal analysis	
Model	AUC	F1-score	AUC	F1-score	AUC	F1-score
Clinical Features	0.79	0.74	0.77	0.67	0.70	0.60
Strength	0.62	0.52	**0.86**	**0.77**	0.71	0.61
Betweenness Centrality	0.73	0.68	0.76	0.64	**0.74**	**0.67**
Clustering Coefficient	0.81	0.76	0.67	0.55	0.73	0.64
*Combined Analysis*	**0.84**	**0.80**	0.72	0.61	0.67	0.54

### 2.5 MRI acquisition

Between 2009 and 2013 (N = 27) patients were scanned on a 3 T GE Signa Excite HDx (Taylor et al., 2018). Single-shell dMRI data were acquired using a cardiac-triggered single-shot spin-echo planar imaging sequence (Wheeler-Kingshott et al., 2002): 1.875 × 1.875 × 2.4 mm resolution, gradient directions: 6 and 52 at b-values: 0 and 1200/ mm^2^, δ/Δ/TE = 21/29/73 ms, and a 3D T1-weighted sequence was acquired. For verbal fluency fMRI (Tombaugh et al., 1999) gradient-echo planar T2*-weighted images were acquired with 58 contiguous 2.5 mm oblique axial slices, 96 × 96 matrix reconstructed to 128 × 128 for an in-plane resolution of 1.875 × 1.875 mm (TE/TR = 25/2500 ms).

Between 2014 and 2019 (N = 17) patients were scanned on a 3 T GE Discovery MR750 (Taylor et al., 2018). A 3D T1-weighted sequence (MPRAGE) was acquired and multi-shell dMRI (2 mm isotropic resolution, gradient directions: 11, 8, 32, and 64 at b-values: 0, 300, 700, and 2500 s/mm^2^; ∂/Δ = 21.5/35.9 ms, TE/TR = 74.1/7600 ms). For verbal fluency fMRI (Tombaugh et al., 1999) gradient-echo planar T2*-weighted images was acquired with 50 contiguous 2.4 mm (0.1 mm gap) slices with a 24 cm field of view, 64 × 64 matrix with an in-plane voxel size of 3.75 × 3.75 mm (TE/TR = 22/2500 ms).

### 2.6 MRI processing

#### 2.6.1 Diffusion processing

Diffusion MRI data were denoised (Veraart et al., 2016), Gibbs-unringed (Kellner et al., 2016), corrected for signal drift (Vos et al., 2017), and distortion corrected using a synthesized b0 for diffusion distortion correction (Synb0-DisCo) (Schilling et al., 2019) with FSL topup (Andersson et al., 2003). Eddy currents and movement artifacts were corrected (Andersson and Sotiropoulos, 2016), rotating the b-vectors (Leemans and Jones, 2009). Additionally, bias-field correction was performed in MRtrix3 (Kellner et al., 2016). Response functions for cerebrospinal fluid, white and grey matter were estimated using Single-Shell 3-Tissue (Dhollander and Connelly, 2016) and Multi-Shell 3-Tissue (Dhollander et al., 2019) CSD in MRtrix3 (Kellner et al., 2016).

#### 2.6.2 fMRI processing

Functional MRI data was used to determine patients’ expressive language lateralisation for the inclusion criteria in this study. Each patient performed a verbal fluency task-based fMRI language task. This consisted of a block design with 30 s of covert object generation beginning with the letter presented on screen alternating with 30 s of cross-hair fixation for the baseline condition over 5.5 min (Powell et al., 2006). Hemispheric language lateralization was calculated using the bootstrap method of the lateralization index toolbox implemented in SPM8 (Wilke and Lidzba, 2007) on spmT maps. The WFU PickAtlas’ anatomical masks of the middle and inferior frontal gyri (including the pars triangularis, orbitalis, opercularis) were used based on previous research highlighting lateralising reliability of these regions (Szaflarski et al., 2017, Woermann et al., 2003). LI values were calculated: [LI=(L–R)/(L + R)].

#### 2.6.3 Resection mask

Resection masks were drawn based on previous techniques (Taylor et al., 2018). Post-operative T1-weighted MRI were affinely registered to pre-operative T1-weighted MRI. Resection masks were then manually drawn in MRtrix3 by overlaying the post-operative T1-weighted MRI on the pre-operative T1-weighted MRI starting at the most anterior coronal slice of the temporal lobe, then proceeding posteriorly every three slices. Coronal slices were then joined by drawing in every sagittal slice. Masks were saved in pre-operative T1-weighted space. Resection mask reliability and validity were assessed via inter-rater reliability between two raters. Impact of delineation accuracy was assessed using dilated resection masks (eAppendix 1, eTable 1).

#### 2.6.4 Network Generation

Anatomically constrained tractography (ACT) (Smith et al., 2012) using hybrid surface and volume segmentation in MRtrix3 (Smith et al., 2020) was performed using second-order integration over fiber orientation distribution probabilistic fiber tracking algorithm (Tournier et al., 2010). Tractography was seeded on the boundary of white and grey matter and selecting 10 million streamlines. Spherical-deconvolution informed filtering of tractograms (SIFT) (Smith et al., 2013) was performed filtering tractograms down to 1 million streamlines. This served as our preoperative connectivity. To infer estimated postoperative connectivity, we removed all tracts that passed through the resection mask.

The automated anatomical labelling atlas 2 (AAL2) (Rolls et al., 2015) was transformed to native space by registering the brain MNI to the native space brain (which was extracted using SynthStrip (Hoopes et al., 2022) using non-linear registration in NiftiReg (Modat et al., 2014, Modat et al., 2010). This included 94 cortical regions. To verify consistency across parcellations we also generated network metrics using the Harvard-Oxford cortical and subcortical structural atlas (114 cortical regions) which can be seen in eAppendix 2 (eTable 2). Connectivity matrices were generated by assigning tract endpoints within 1 mm to each subcortical and cortical parcel. Connections from a region to itself were removed. As graph theory metrics can vary across the level of threshold applied, a threshold and average were applied similar to Bassett et al. (Bassett et al., 2008). Matrices were thresholded based on the prevalence of a connection across patients. This threshold increased in increments of 5%, from 75% to 100%. This was done to increase the reliability of connections included. Graph metrics were then averaged across thresholds. Connectivity matrices were transformed using a log_10_ transformation due to the distributions being non-normal (see eAppendix 3).

### 2.7 Network quantification

Network metrics (graph theory metrics) were generated using weighted and undirected versions of functions in the Brain Connectivity Toolbox (Rubinov and Sporns, 2010). Network metrics were generated taking into account the entire brain. We investigated measures of strength, betweenness centrality, and clustering coefficient.

#### 2.7.1 Cortical Strength

**Fig. 1:**
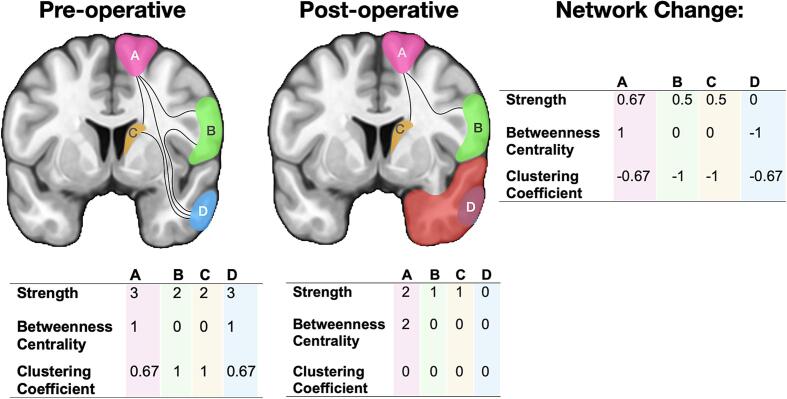
A visual representation of the pre-operative and post-operative network and network metrics: strength, betweenness centrality and clustering coefficient. Each line represents one streamline. Each coloured cortical region (pink, green, yellow, blue) represent a hypothetical cortical region. Red represents the resection mask. (For interpretation of the references to colour in this figure legend, the reader is referred to the web version of this article.)

Cortical strength represents the sum of white matter connections to/from a cortical region, encapsulating the number of connections a given region has (Fig. 1.). The decrease of white matter connecting a cortical region gives an approximation of how the resection has impacted on cortical regions’ connectivity involved in the network. Estimated change in strength was calculated by dividing post-operative by pre-operative values, because post-operative values should never increase.

#### 2.7.2 Betweenness Centrality

The betweenness centrality of a region represents the number of times a cortical region is a point on the shortest path between two other cortical regions (Fig. 1.). Betweenness centrality has been related to the speed of information processing (Krukow et al., 2019) and provides a metric of how important that node is in transfer of information. Estimated change in betweenness centrality were calculated by subtracting the post-operative by the pre-operative values due to their ability to increase or decrease following resection (Taylor et al., 2018).

#### 2.7.3 Clustering coefficient

The clustering coefficient provides information on the likelihood that neighbours of a given cortical region are interconnected with each other (Fig. 1.). It can be interpretated as how tightly knit the surrounding network is. It is associated with efficient information exchange (Sporns et al., 2004). Similar to betweenness centrality, the clustering coefficient can increase following surgery, thus estimated change is calculated by subtracting the post-operative by the pre-operative values (Taylor et al., 2018).

### 2.8 Analysis

In order to assess the classification capability of clinical features, and the estimated change from pre- to post-operative graph theory metrics (strength, betweenness centrality, and clustering coefficient) both individually and as a group to binarized (via the RCI) picture naming decline at 3 and 12 months we used an established machine learning framework (Taylor et al., 2018).

#### 2.8.1 Machine learning

To produce unbiased feature selection in assessing picture naming decline we used a two-step leave-one-out cross validation and feature selection method (Taylor et al., 2018). This resulted in feature selection being independent of the test dataset.

Depending on the prevalence threshold, the range of missing connections per cortical region ranged between 25% and 0%. Each feature was harmonised across scanners using NeuroCombat performing empirical Bayes harmonization across features with parametric adjustment (Fortin et al., 2017). The data were then scaled to a standard deviation of 1.

Feature (variable) selection was performed using a penalized least squares approach with a smoothly clipped absolute deviation (SCAD) penalty function (Fan and Li, 2001) with integrated leave-one-out cross validation (splitting data into 43 training subjects and 1 test subject, 44 times). This was performed to select variables unbiased by the testing dataset, resulting in 44 different models being trained (Becker et al., 2011). With this approach, the coefficient β are those that minimise the function:

(1) $$J\left(\beta \right)=\frac{1}{2n}{|\left|y-X\beta \right||}_{2}^{2}+\sum_{j=1}^{p}p\left(\beta_{j},a,\lambda \right)$$

where:

(2) $$p\left(\beta_{j},a,\lambda \right)=\left\{\begin{array}{c} \lambda \left|\beta \right|\left|\beta \right|\leq \lambda ;\\ \frac{2a\lambda \left|\beta \right|-\beta^{2}-\lambda^{2}}{2(a-1)}\lambda <\left|\beta \right|\leq a\lambda ;\\ \frac{\lambda^{2}\left(a+1\right)}{2}\left|\beta \right|>a\lambda . \end{array}\right)$$

Here, *a* represents the concavity parameter for SCAD, which was varied 50 times with values evenly spaced between 2 and 10. The λ was varied 100 times with a minimum ratio of 0.5, the maximum value was the minimum regularisation parameter which yielded an all-zero estimate. This resulted in 5000 unique gamma and lambda combinations for each leave-one-out model. SCAD was chosen due to its superior performance in feature selection (Becker et al., 2011) and the expectation of relatively large coefficients.

To assess the non-zero features for each leave-one-out cross validation remaining after each λ and *a* penalisation on picture naming decline, we used a linear support vector classifier incorporating a leave-one-out cross-validation scheme. This two-step machine learning method allowed feature selection and support vector classification unbiased from the testing dataset. Due to class imbalances (3 months: 17/27; 12 months: 11/26; decline/no decline) for each leave-one-out iteration, we incorporated synthetic minority over-sampling technique (SMOTE) (Chawla et al., 2002) on the training dataset. To increase speed and reduce complexity of the models tested, only models under 20 variables were trained / tested in the support vector classification. While this does not guarantee the final model will have a maximum of 20 variables as a result of the two-step leave-one-out cross validation and feature selection, it does reduce the number of models and variables tested. Trained feature coefficients were extracted to signify their importance in the model. The resulting model was then tested on the patient left out of the training set, with the patient’s predicted and actual outcome saved.

To select the best model and the best feature combination across each *a* and λ (from SCAD) we used the receiving operator characteristic ‘area under curve’ (AUC), selecting the maximum score. The AUC represents the ability of a classifier to distinguish between classes, a bigger value represents better proportion between true and false positive rate. The F1-score was also calculated as a measure of accuracy from the precision and recall, where precision is the amount of true positive predictions divided by total number of positive predictions (true positive + false positive), and recall is the amount of the true positive predictions divided by the total number of actual positive observations (true positive + false negative).

The reported models may have different features in each leave-one-out iteration (due to incorporating leave-one-out cross-validation in our feature selection to remove bias) and each threshold. To accurately describe feature importance, we created the weighted feature importance metric. This averaged the mean importance across all leave-one-out and threshold iterations. The feature importance was weighted with the percentage of inclusion to identify the most important feature across models/thresholds. This was done to avoid skewing results with features which were important but only occurring in one model.

#### 2.8.2 Longitudinal support vector classifier

To assess if there were features which were able to classify patients who underwent RCI determined decline at 3 months and who then experienced further decline between 3 and 12 months, we employed a two-step linear support vector classifier classification chain. This first aims to correctly classify patients who underwent decline at 3 months, then the features and result of this classification are used to predict if patients will then undergo further decline by 12 months. Feature selection was performed twice as described above, using both the 3- and 12-month RCI decline. As SMOTE is unavailable for multioutput datasets we addressed class imbalances by weighting classes based on their proportion of the sample. As feature importance can no longer be identified from this model, we instead recorded the prevalence of features in each leave-one-out classification model, expecting those with the highest prevalence to be the most important. To select the best feature combination, we calculated a weighted AUC, which calculated the AUC separately for each timepoint, and then combining the results weighting by the number of true instances for each label.

#### 2.8.3 Statistical analysis

We used permutation testing as in Gleichgerrcht et al. (Gleichgerrcht et al., 2020) to assess if model prediction was significantly better than chance. Based on the final model, variables were extracted and underwent SMOTE. 10,000 permutations were then used, shuffling the picture naming RCI and splitting the data into train and test sets. A linear support vector classifier was trained on the training data and used to predict the test data. The AUC for each permutation was calculated. The p-value was calculated based on the number of times the AUC was higher in the permutation model compared to the main model.

Similarly, we used permutation testing to assess differences between those with and without picture naming decline in metrics selected by the machine learning model. For each variable, data were split by the picture naming RCI and a Welch’s *t*-test was performed on the actual data to calculate the actual T-value. For permutation testing (10,000 permutations) the RCI was shuffled, and each variable data were split by the reshuffled RCI, calculating the T-value. The p-value was calculated based on number of times the T-value in each variable was higher in the permutation model compared to the actual T-value. The mean p-value was calculated for each variable across thresholds and Bonferroni correction to account for multiple comparisons was applied.

## 3 Results

### 3.1 Machine learning overview

The results for classifying binarized (via the RCI) picture naming decline at 3- and 12- months are summarised in Table 1. At 3 months, the best performing metric was a combination of network metrics and clinical features. At 12 months, the best performing metric was the estimated change in strength to cortical regions. Looking at metrics classifying decline at 3 months and using that information to classify decline at 12 months, estimated betweenness centrality change was the best metric in classifying picture naming decline.

### 3.2 Picture naming 3 months: Combined analysis

The best performing model was a combined analysis, including 26 features. A permutation-based comparison of this combined model with a random model gave an AUC of 0.84 for the former and AUC = 0.50 for the latter (*p* < 0.001). This equated to a specificity of 85.2% and sensitivity of 77.8%, with an overall accuracy of 84.1%. This translates to correctly identifying 14/17 with and 23/27 without picture naming decline. When comparing this to the Harvard-Oxford cortical and subcortical structural atlas (see eAppendix 2, eTables 2) we demonstrate similar results with combined analysis being the best predictor with an AUC of 0.81 and an F1-score of 0.77.

**Fig. 2:**
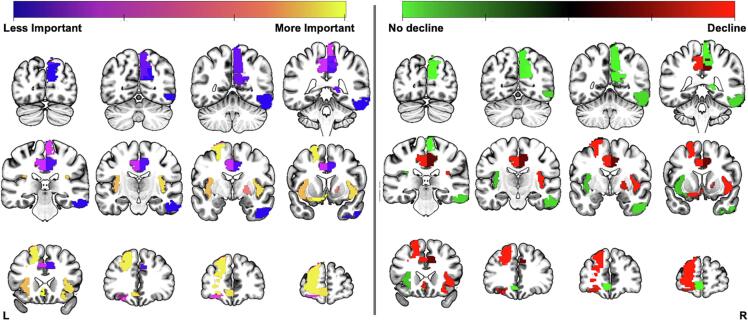
A mosaic of the most 15 important cortical regions included across all thresholds in the 3 months combined analysis classification as described in Table 3. The left panel illustrates weighted feature importance. The right panel shows if the metric was higher for patients with (red) or without (green) picture naming decline. (For interpretation of the references to colour in this figure legend, the reader is referred to the web version of this article.)

Table 3 summarises 15 of the most important features included in the models. This included 7 betweenness centrality, 5 clustering coefficient, 1 strength, and 2 clinical features with 6 being ipsilateral to the resection (see Fig. 2). The most important feature was the estimated change of clustering coefficient in the left sub-callosal anterior cingulate cortex.

**Table 3:** 15 of the most important features (as defined by weighted importance) to the combined model classification across leave-one-out in a support vector classification model for inferring 3 months picture naming decline. Showing the weighted importance across leave-one-out models, each cortical region and the associated graph metric contributing significantly to the model, the corrected p-value for a permutation test (10,000 permutations, Bonferroni corrected), and the mean difference with a higher value representing that metric was higher in those with picture naming decline. For each region, the lobule is shown in brackets next to the region name.

Weighted Importance	Region	Metric	*p*-values	Mean-difference
76.91	Left Sub-callosal Anterior Cingulate Cortex (SC)	Clustering Coefficient	0.001	0.76
66.85	Left Dorsolateral Superior Frontal Gyrus (F)	Clustering Coefficient	0.001	0.9
64.93	Left Superior Frontal Gyrus Medial Orbital (F)	Betweenness Centrality	0.002	−0.85
64.37	Right Insula (I)	Clustering Coefficient	0.004	0.79
61.23	Left Insula (I)	Clustering Coefficient	0.001	−0.54
50.51	Right Pallidum (SC)	Betweenness Centrality	0.002	0.54
30.99	Pre-operative Picture Naming Scores	Clinical	0.002	0.85
27.59	Left Anterior Orbitofrontal Cortex (F)	Betweenness Centrality	0.002	0.57
22.95	Right Paracentral Lobule (P)	Betweenness Centrality	0.002	−0.75
17.51	Left Middle Cingulate (SC)	Betweenness Centrality	0.002	0.69
14.77	fMRI Lateralisation Index	Clinical	0.002	1.03
9.73	Right Precuneus (P)	Betweenness Centrality	0.002	−0.64
7.98	Right Middle Cingulate (SC)	Strength	0.002	0.32
3.52	Right Inferior Temporal Gyrus (T)	Betweenness Centrality	0.002	−0.58
2.89	Right Cuneus (O)	Clustering Coefficient	0.001	−0.76

Abbreviations: F: frontal; I: Insula; O: Occipital; P: parietal; SC: subcortical; T: temporal.

The anatomical distribution of classification importance for picture naming decline at 3 months can be seen in the left panel in Fig. 2. The right panel shows the mean difference between patients with and without picture naming decline. Red represents the mean difference was greater for patients with decline while green represents it was greater for patients without picture naming decline.

### 3.3 Picture naming 12 Months: Strength

The best performing model was the estimated change in strength following surgery, including 11 features. Using a permutation test to compare how this model performed against a random model, an AUC of 0.86 was significantly higher than random (random model AUC = 0.50, *p* = 0.002). This equated to a specificity of 80.77% and sensitivity of 66.67%, with an overall accuracy of 83.78%. This translates to correctly identifying 10/11 with and 21/26 without picture naming decline. When comparing this to the Harvard-Oxford cortical and subcortical structural atlas (see eAppendix 2, eTables 2) we demonstrate different results. The strength metric of this atlas had an AUC of 0.57 with an F1-score of 0.45. Rather, betweenness centrality was the best predictor which showed similar metrics as presented here (AUC = 0.72, F1-score = 0.61).

**Fig. 3:**
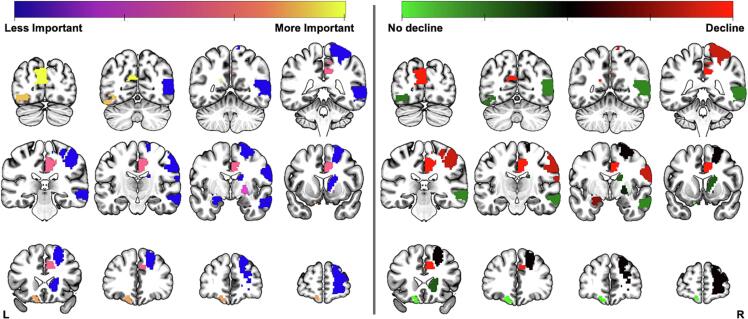
A mosaic of the 6 cortical regions included across all thresholds in the 12 months combined analysis classification as described in Table 4. The left panel illustrates weighted feature importance with yellow (more) and purple (less) representing how important that feature was for classification. The right panel shows if the metric was higher for patients with (red) or without (green) picture naming decline. (For interpretation of the references to colour in this figure legend, the reader is referred to the web version of this article.)

Table 4 summarises the 11 features included in the models. 4 variables were ipsilateral to the resection (see Fig. 3). The most important feature was the estimated change in strength to the left cuneus.

**Table 4:** The 11 features (ordered by weighted importance) involved in the estimated change in strength model classification across leave-one-out in a support vector classification model for inferring 12 months picture naming decline. Showing the weighted importance across leave-one-out models, each cortical region and the associated graph metric contributing significantly to the model, the corrected p-value for a permutation test (10,000 permutations, Bonferroni corrected), and the mean difference with a higher value representing that metric was higher in those with picture naming decline. For each region, the lobule is shown in brackets next to the region name.

Weighted Importance	Region	Metric	*p*-values	Mean-difference
134.66	Left Cuneus (O)	Strength	0.001	0.44
108.52	Left Inferior Occipital Gyrus (O)	Strength	0.001	−0.63
103.8	Left Orbito-frontal Cortex, Medial (F)	Strength	0.001	−1.14
77.97	Right Cingulate, Middle (SC)	Strength	0.001	0.67
51.05	Right Pallidum (SC)	Strength	0.001	−0.39
25.33	Right Anterior Cingulate (SC)	Strength	0.001	0.42
2.32	Right Postcentral Gyrus (P)	Strength	0.001	0.28
2.25	Left Amygdala (T)	Strength	0.002	0.14
1.77	Right Caudate (SC)	Strength	0.001	−0.56
1.05	Right Superior Frontal Gyrus, Dorsolateral (F)	Strength	0.001	−0.17
0.59	Right Middle Temporal Gyrus (T)	Strength	0.001	−0.68

Abbreviations: F: frontal; O: Occipital; P: parietal; SC: subcortical; T: temporal.

The anatomical distribution of classification importance for inferring picture naming decline at 12 months can be seen in the left panel in Fig. 3. The right panel shows the mean difference between patients with and without picture naming decline. Red represents the mean difference was greater for patients with decline while green represents it was greater for patients without picture naming decline.

### 3.4 Picture Naming: Longitudinal analysis

The best performing model in classifying picture naming outcome at 3 months and using these results to classify 12 months outcome was the estimated change in betweenness centrality following surgery, this included 25 features. Using a permutation test to compare how this model performed against a random model, an AUC of 0.74 was significantly higher than random (random model AUC = 0.50, *p* = 0.001). This equated to a sensitivity of 63.6% and 44.4% and a specificity of 63.6% and 61.5% for 3- and 12-months classification, respectively, with an overall accuracy of 60%. This translates to correctly identifying 14/15 and 8/11 of patients with decline and 14/22 and 16/26 without decline at 3- and 12-months, respectively. These are similar metrics to those from the Harvard-Oxford cortical and subcortical structural atlas (see eAppendix 2, eTables 2). The betweenness centrality metric had an AUC of 0.66 with an F1-score of 0.57. However, the combined analysis produced a better AUC for this atlas (AUC = 0.79, F1-score = 0.72).

**Fig. 4:**
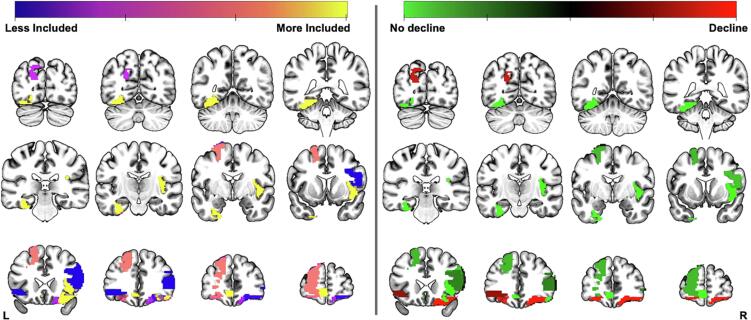
A mosaic of the 15 cortical regions included across all thresholds across the chained 3- and 12-months betweenness centrality analysis as described in Table 5. The left panel illustrates feature inclusion with yellow (more) and purple (less) representing the percentage of inclusion in each leave-one-out cross validation model. The right panel shows if the metric was higher for patients with (red) or without (green) picture naming decline*.* (For interpretation of the references to colour in this figure legend, the reader is referred to the web version of this article.)

Table 5 summarises 15 of the most important features included in the model, based on feature inclusion (see Fig. 4.). Six of these variables were ipsilateral to the resection. The most important feature was change in betweenness centrality to the right insula.

**Table 5:** 15 of the most important features (as defined by weighted importance) to the combined model classification across leave-one-out in a chained support vector classification model for inferring 3- and then 12-months picture naming decline. As variable importance was unable to be extracted, we used the percentage of inclusion in leave-one-out models to judge the importance of cortical regions. We show the importance, each cortical region and the associated the graph metric contributing significantly to the model, the corrected p-value for a permutation test (10,000 permutations, Bonferroni corrected), and the mean difference with a higher value representing that metric was higher in those with picture naming decline. For each region, the lobule is shown in brackets next to the region name.

Percentage Inclusion	Region	Metric	*p*-values	Mean-difference
100	Right Insula (SC)	Betweenness Centrality	0.002	−0.69
100	Left Fusiform Gyrus (T)	Betweenness Centrality	0.002	−0.64
97.3	Left Superior Frontal Gyrus Medial Orbital (F)	Betweenness Centrality	0.003	−0.65
97.3	Right Posterior Orbitofrontal Cortex (F)	Betweenness Centrality	0.001	0.65
94.59	Right Lateral Orbitofrontal Cortex (F)	Betweenness Centrality	0.002	−0.64
70.27	Left Dorsolateral Superior Frontal Gyrus (F)	Betweenness Centrality	0.001	−0.44
62.16	Left Anterior Orbitofrontal Cortex (F)	Betweenness Centrality	0.002	0.63
24.32	Left Superior Occipital Gyrus (O)	Betweenness Centrality	0.002	0.37
21.62	Right Medial Orbitofrontal Cortex (F)	Betweenness Centrality	0.002	0.57
8.11	Right Anterior Orbitofrontal Cortex (F)	Betweenness Centrality	0.002	0.51
2.7	Right Pars Opercularis (F)	Betweenness Centrality	0.002	−0.46
2.7	Right Pars Triangularis (F)	Betweenness Centrality	0.003	−0.34
2.7	Left Orbitalis (F)	Betweenness Centrality	0.001	0.28
2.7	Left Rectus (F)	Betweenness Centrality	0.002	0.4
2.7	Left Posterior Cingulate (SC)	Betweenness Centrality	0.002	−0.63

Abbreviations: F: frontal; I: Insula; O: Occipital; SC: subcortical; T: temporal.

## 4 Discussion

We combined clinical information with structural brain network measures to infer their relationship with naming outcome following ATLR. We demonstrate that, for picture naming at 3 months, a combination of clinical and network metrics including strength, betweenness centrality, and clustering coefficient can infer naming decline with high accuracy. We also showed that for picture naming at 12 months, estimated change in strength had high classification accuracy. Longitudinal analyses demonstrated that betweenness centrality was the best metric for classifying patients at 3 months and then for assessing further decline at 12 months. While previous work has highlighted pre-operative structural network measures associated with pre-operative performance (Munsell et al., 2019), we demonstrate the classification utility of the estimated change in network metrics with immediate and longer-term post-operative picture naming decline.

Previous research indicated the role of clinical characteristics in predicting which patients will undergo naming decline (Busch et al., 2018). We support the notion that clinical characteristics are predictive of 3 month post-operative naming decline. Higher pre-operative picture naming scores were predictive of a worse outcome following surgery. Patients with higher cognitive function have “more to lose” (McAndrews and Cohn, 2012). Older age at epilepsy onset was predictive of worse outcome, as has been found previously (Busch et al., 2016). For 12 month and longitudinal post-operative naming decline, estimated change in strength and betweenness centrality, respectively, alone outperformed clinical characteristics and other networks combined. While we did not replicate the same predictive capability of clinical features as in Busch et al. (Busch et al., 2018), the difference between our and their study population could account for this. We included only those having language-dominant ATLR, which is already an “enriched” population at risk of decline, and was a strongly predictive variable in Busch et al. (Busch et al., 2018).

Language representation in TLE is atypical as reorganisation disperses function to similar contralateral regions, ipsilateral regions involved in language take on a greater role, as well as regions that are not typically involved in language (Binding et al., 2022). This could explain why contralateral and sub-cortical regions were so prominent, across all analyses. This could also explain why classical language regions such as the ipsilateral temporal lobes (Binding et al., 2022) did not appear important.

Picture naming is a complex cognitive function that involves the coordinated activity of multiple cortical regions dispersed throughout the brain. Research using graph theory to analyse the structural connectome has revealed that the communicability of these regions is essential for successful naming, and dysfunction can impair language function in patients with TLE (Balter et al., 2019). These findings demonstrate that preoperative naming performance can be predicted to a high degree by analysing graph theory metrics of the structural connectome in TLE patients. Our study found that estimated changes in all graph theory metrics (i.e., clustering coefficient, betweenness centrality, and strength) and clinical factors were able to classify decline in picture naming at 3 months after ATLR with an AUC of 0.89. Previous research showed that language function dips immediately after surgery followed by some patients improving (Binding et al., 2023). As such, our results suggest that all clustering coefficient, betweenness centrality, strength, and clinical factors play a role in the preparedness of the brain to a temporal lobe resection.

Estimated changes in strength to specific cortical regions had the best performance in classifying picture naming outcome at 12 months. Specifically, our analysis identified the cuneus, inferior occipital gyrus, and orbito-frontal cortex as being strongly associated with decline. It is of interest that a post-operative increase in cuneus connection strength was associated with better outcome, while decreases in strength were important for the inferior occipital and orbito-frontal gyrus – indicating the complexity of the brain network changes and functional dependence on the network. The mechanisms underlying the cuneus estimated changes are not clear and may reflect a secondary effect with the removal of abnormally functioning regions that were interfering with picture naming network function. Further work to investigate these results would be essential to understand the mechanism behind the observed picture naming improvement. Greater resection of connections to the inferior occipital gyrus and orbito-frontal cortex was associated with patients with picture naming decline. The inferior occipital gyrus is part of the ventral visual processing pathway, which is essential for object recognition and visual association (Ungerleider, Epub 1982,, Creem and Proffitt, 2001). The medial orbito-frontal cortex is implicated in successful memory encoding and retrieval (Duarte et al., 2010). Reduced white matter connectivity between these regions could impair visual recognition and memory retrieval. We can infer that this supports our previous research which linked the integrity of the inferior-fronto-occipital fasciculus (IFOF), which interconnects these regions, to picture naming decline at 3 months post-injury (Binding et al., 2023). While our previous analysis did not find a relationship between IFOF integrity and naming decline at 12 months, the IFOF encapsulates cortical connections to many regions beyond the two regions we included in our analysis.

When investigating which network metric was the best at classifying outcome at 3-, and then 12-months in our longitudinal analysis, we found that betweenness centrality outperformed other metrics and combinations. This could be due to betweenness centrality changes to specific cortical regions being more detrimental to post-operative functional reorganisation. The features with the strongest inclusion were the right insula, left fusiform gyrus, left superior frontal gyrus and right orbitofrontal cortex regions. Increased fMRI activity within the insula is proposed to be related to difficulty in articulatory effort (Sherman et al., 2011). Decrease of integration of the insula, failing to convey the correct articulatory motor movements with the surrounding network could lead to expressive errors or the “tip of the tongue” effect (Hamberger, 2003). The fusiform gyrus shows high specialisation to visual naming tasks, a disconnection of this region from the surrounding network could inhibit visual discrimination of objects (Binding et al., 2022). The orbitofrontal cortex is hypothesised to be mainly related to memory, with its function being implicated in learning and reversing associations (Rolls, 2004). This highlights the multifaceted function of picture naming, and language in general, where reductions in any one of the associated functions in an interconnected network can lead to an impairment in ability.

### 4.1 Clinical utility

**Fig. 5:**
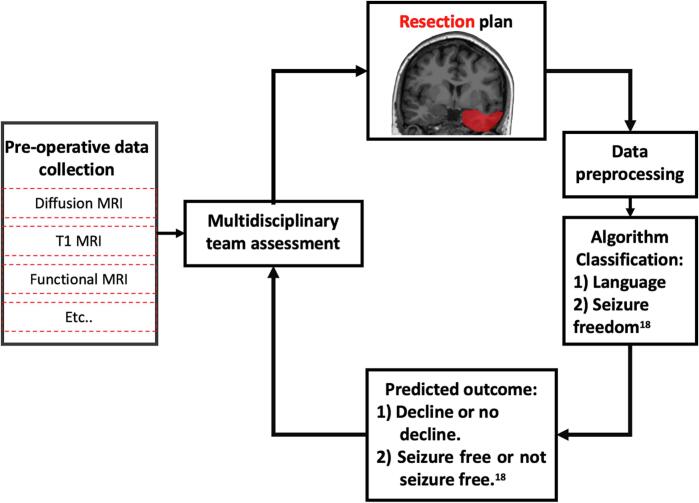
Adapted from Taylor et al.(Taylor et al., 2018) The combined clinical utility of the algorithms produced in this paper and in Taylor et al.(Taylor et al., 2018) From left to right: Pre-operative MRI data is acquired and evaluated at multidisciplinary team meetings. If surgery is recommended data is a planned resection cavity is drawn and the data are pre-processed, using the resection mask to extract the expected change in network metrics. The estimated change in metrics is then fed into ours and Taylor et al.(Taylor et al., 2018) algorithms to infer if the patient will be seizure free and if they will undergo picture naming decline. This information could be used to modify the resection plan to minimise the impact on network measures while maximising seizure freedom chances. The final plan could be used to inform the patient of the expected risk and remission rates.

The language network is complex and widespread in healthy brains. In TLE, this network is atypical with function being dispersed to new regions or regions attaining new functions (Binding et al., 2022). Our retrospective analysis of estimated network changes using pre- and post-operative data might be used in the future to prospectively predict cognitive outcomes. In line with previous suggestions (Taylor et al., 2018), pre-operative whole-brain tractography can be performed and an intended resection drawn based on pre-operative structural T1-weighted scans. This could be combined with previous work (Taylor et al., 2018) to identify the optimal resection cavity for seizure freedom and cognitive outcome. An example of the clinical workflow is demonstrated in Fig. 5, which shows how these could be combined to maximise patient outcome. To reduce the impact on the structural network, more limited resections could be used as research shows no relationship between resection size and seizure outcome (Binding et al., 2023, Galovic et al., 2019). Further, the use of laser interstitial thermal therapy (LITT) for ablations could reduce the footprint of the surgery, limiting the impact on the network (Kohlhase et al., 2021).

### 4.2 Research evaluation

All patients included in this study had surgery performed by the same two surgeons (AM, AMc). This had the benefit of ensuring there was a consistent surgical approach for all cases. However, replication studies are required to assess the generalizability of our findings to other centers.

The use of manually-drawn resection masks to estimate post-operative tractography has the benefit of the rater being able to visually estimate for brain-shift but may introduce human error and image registration issues. Additional analyses were performed to investigate these issues and showed minimal impact (eAppendix 1).

Several steps were taken to ensure the accuracy of our methods. As for tractography: (i) it was seeded on the boundary of grey and white matter boundary; (ii) we used ACT to ensure tractography could only run through white matter; (iii) we used SIFT to filter down probabilistic-tractography; and (iv) we thresholded the connectome based on the presence of the connection across individuals, making our results more reproducible. These steps increased the accuracy and replicability of our results. However, whole-brain tractography still comes with inherent problems such as false positives (Maier-Hein et al., 2017). Furthermore, results will depend on data quality, for which we include two levels, single- (poor) and multi- (good) shell data. We attempted to addressed this using NeuroCombat, normalising the two scanners output, but this does not change the inherent difference in the diffusion quality.

The current study identified various cortical regions whose estimated change in network metrics were associated with picture naming decline. For prospective use, a proposed resection cavity mask could be used to generate estimated changes in graph theory metrics to determine whether this could infer decline in picture naming. Further research is needed to investigate if pre-operative graph metrics alone can accurately predict patient decline, thereby increasing the applicability of this approach.

While we performed cross validation with regard to prediction models, we were unable to further split data into training, testing, and validation sets because of a limited sample size. Our 12 month analysis was already limited by a reduced sample size which could reflect the increased classification ability. Additional analysis with the validation cohort would ensure unbiased model evaluation and aid in mitigating issues such as overfitting, where the model may perform well on the training data, but poorly on new data. Future research should aim to expand the sample size to permit a validation dataset.

We investigated whether network metrics remained stable in classifying outcome across different atlases (eAppendix 2). We found that while the overall classification ability remained the same at each timepoint, the network metric of importance varied. This could be due to differences in cortical anatomy between atlases, for example, the Harvard-Oxford atlas splits each temporal gyri into anterior, middle, and posterior portions. Nevertheless, application of these results should be done with the same methodology described above for maximum reliability.

### 4.3 Future directions

Language representation is abnormal in TLE, and there are varying patterns of reorganization. This variability could contribute to the high degree of variability observed in leave-one-out models, in which the inclusion or exclusion of individual patients can affect the classification performance of network metrics. To improve classification accuracy, it may be useful to incorporate fMRI to map functional reorganization on a patient-specific level. Combining fMRI with dMRI to map the structural network could provide a more comprehensive understanding of the interplay between functional reorganization and structural connectivity. This approach could lead to more accurate classification of language dysfunction following surgery and ultimately inform targeted interventions to preserve language function.

While we have limited the scope of this paper to ATLR and naming decline, this method could be used in other epilepsy surgeries, such as frontal lobe epilepsy, in which language is impacted differently from TLE (Caciagli et al., 2021). Additionally, change in other cognitive domains in TLE may be better predicted using these metrics, such as post-operative memory change (Fleury et al., 2022).

This research could be utilised for pre-operative interventions to preserve cognitive function. Our results highlight the involvement of regions away from the resection site. Transcranial magnetic stimulation has demonstrated it can be used to induce suppression of the semantic network and upregulation of compensatory regions (Binney and Ralph, 2015). This could be used on cortical regions included in the model to alter activity and the picture naming network. Further fMRI research is required to corroborate these results and the activity seen at each cortical region.

The present study relies on the use of resection masks that were manually drawn. Although our results show a considerable degree of consistency in the definition of the resection cavity across different delineators (as detailed in eAppendix 1), it is imperative that future research explores the impact of minor modifications to the boundaries of these masks on the stability of graph theory metrics. Such investigations would have valuable implications for the clinical utility of this approach, as they would provide insights into the precision required for pre-clinical assessments of resection masks.”.

## 5 Conclusion

The estimated changes in network metrics following language-dominant ATLR can classify the picture naming outcome of patients with high accuracy at both 3- and 12- months post-operatively. We also highlight cortical regions in which change in betweenness centrality is related to picture naming outcome at 3 months and then at 12 months. This method could be used to improve the information available to patients about their risk of naming decline from surgery and be utilised in resection planning to minimise the impact on the wider network.

## Study funding

Authors Lawrence P. Binding and Sjoerd B. Vos are supported by Epilepsy Research UK (grant number P1904). Authors John S. Duncan receive funding from the Wellcome Trust Innovation Program (218380/Z/19/Z) and Epilepsy Research UK (grant number P1904). Lawrence P. Binding, Sjoerd B. Vos, and John S. Duncan are partly funded by the National Institute for Health Research University College London Hospitals Biomedical Research Centre (NIHR BRC UCLH/UCL). Peter Taylor is supported by a UKRI Future Leaders Fellowship (MR/T04294X/1). Gavin Winston was supported by the Medical Research Council (G0802012, MR/M00841X/1). FX was supported by a Newton International Fellowship of the Academy of Medical Sciences and the Newton Fund (NIF\R5\264) and supported by Wellcome trust (221934/Z/20/Z). Gavin Winston and John Duncan were supported by the medical research council / Wellcome Trust between the years of 2008–2013 (MRCG0802012 / 083148).

## CRediT authorship contribution statement

**Lawrence Peter Binding:** Conceptualization, Formal analysis, Investigation, Methodology, Visualization, Writing – review & editing. **Peter Neal Taylor:** Conceptualization, Methodology, Formal analysis, Writing – review & editing, Funding acquisition. **Aidan G. O'Keeffe:** Methodology, Writing – review & editing. **Davide Giampiccolo:** Writing – review & editing. **Marine Fleury:** Writing – review & editing. **Fenglai Xiao:** Writing – review & editing. **Lorenzo Caciagli:** Writing – review & editing, Methodology, Data curation. **Jane de Tisi:** Project administration. **Gavin P. Winston:** Data curation, Funding acquisition, Writing – review & editing. **Anna Miserocchi:** Data curation. **Andrew McEvoy:** Data curation. **John S. Duncan:** Supervision, Writing – review & editing, Methodology, Funding acquisition. **Sjoerd B. Vos:** Supervision, Writing – review & editing, Conceptualization, Methodology, Funding acquisition.

## Declaration of Competing Interest

The authors declare the following financial interests/personal relationships which may be considered as potential competing interests: Lawrence P. Binding; Sjoerd B. Vos are supported by Epilepsy Research UK (grant number P1904). John S. Duncan receives funding from the Wellcome Trust Innovation Program (218380/Z/19/Z). Lawrence P. Binding; Sjoerd B. Vos; John S. Duncan are partly funded by the National Institute for Health Research University College London Hospitals Biomedical Research Centre (NIHR BRC UCLH/UCL). Peter Taylor is supported by a UKRI Future Leaders Fellowship (MR/T04294X/1). Gavin Winston was supported by the Medical Research Council (G0802012, MR/M00841X/1). Anna Miserocchi; Aidan O'Keeffe; Jane de Tisi; Andrew McEvoy; Davide Giampiccolo; all report no disclosures relevant to this manuscript.

## Data Availability

Data will be made available on request.
