# Supplementary material for: The impact of temporal lobe epilepsy surgery on picture naming and its relationship to network metric change
Source: Neuroimage Clin. 2023 May 27;38:103444. doi: 10.1016/j.nicl.2023.103444 (PMC10300575; doi:10.1016/j.nicl.2023.103444)
Supplement: Supplementary data 1 [file mmc1.docx]

eAppendix 1. Resection Mask Reliability and Validation

Core to our methodology is accurate resection mask delineation. In order to assess the resection masks validity, we performed interrater reliability assessments based upon 2 raters performed at different times using the same methodology. Stability of our results was based upon dilation of the resection mask. If our results are resection-mask delineation dependent, we should expect to see little correlation with dilation. If our results hold stable in the presence of dilation, this suggests that our results are reliable across any delineation errors.

**eMethod**

*Reliability Assessments*

To assess the reliability of our results, resection masks and volumes were compared between two raters. Resection mask volume of each mask was extracted and we performed two statistical assessments: a two-tailed Pearson’s correlation and a consistency two-way mixed intraclass correlation coefficient.

**eResults**

*Reliability*

All resection masks used in the main manuscript were performed by Rater 1. Rater 2 delineated of a subset of 21 resection masks. Mean and standard deviation of resection volumes are shown in eTable 2. We found a strong significant correlation between resection volume between raters, *r*(21)= 0.840, *p* < 0.001. We also found a strong consistency between the raters: the intraclass correlation coefficient was 0.906 with a 95% confidence interval from 0.769 to 0.962 (F(20,20)=10.652, *p* < 0.001).

*eTable 1. Mean and standard deviation between the overlap of 21 cases drawn by two raters.*

| *Features* | *Rater 1* | *Rater 2* |
| --- | --- | --- |
| Mean (standard deviation) | 36.43 (6.65) | 34.94 (5.61) |
