# Supplementary material for: The impact of temporal lobe epilepsy surgery on picture naming and its relationship to network metric change
Source: Neuroimage Clin. 2023 May 27;38:103444. doi: 10.1016/j.nicl.2023.103444 (PMC10300575; doi:10.1016/j.nicl.2023.103444)
Supplement: Supplementary data 2 [file mmc2.docx]

**eAppendix 2: Atlas comparison**

To ascertain the generalisation of our findings on the AAL2 atlas, we conducted a comparison using the Harvard-Oxford cortical and subcortical structural atlas. The results are presented in eTable2. Our analysis revealed that the combined analysis yielded similar AUCs for a period of 3 months. However, for a 12-month period, the predictive ability of strength was found to be poorer, while betweenness centrality demonstrated similar predictive capability. In summary, our findings suggest that despite the use of different atlases, a consistent trend in predictive capability exists.

*eTable 2. Predictive capability of clinical and graph theory metrics to picture naming decline.*

| Timepoint | 3 Months | | 12 Months | | | Longitudinal analysis | |  |
| --- | --- | --- | --- | --- | --- | --- | --- | --- |
| Model | AUC | F1-score | | AUC | F1-score | AUC | F1-score | |
| Strength | 0.66 | 0.59 | | 0.57 | 0.45 | 0.65 | 0.54 | |
| Betweenness Centrality | 0.65 | 0.62 | | 0.72 | 0.61 | 0.66 | 0.57 | |
| Clustering Coefficient | 0.73 | 0.68 | | 0.51 | 0.41 | 0.69 | 0.62 | |
| *Combined Analysis* | 0.81 | 0.77 | | 0.66 | 0.52 | 0.79 | 0.72 | |
