# Supplementary material for: The impact of temporal lobe epilepsy surgery on picture naming and its relationship to network metric change
Source: Neuroimage Clin. 2023 May 27;38:103444. doi: 10.1016/j.nicl.2023.103444 (PMC10300575; doi:10.1016/j.nicl.2023.103444)
Supplement: Supplementary data 3 [file mmc3.docx]

**eAppendix 3. Additional Statistics**

**Normal Distribution Assessment**

A Kolmogorov-Smirnov test was applied to all 20736 unique cortical connections. For pre- and post-operative data across thresholds, we found that zero of the connections were normally distributed.

**Baseline Values**

To assess if the level of baseline values were related to the change we performed a linear regression on the graph theory metrics pre-operative metric and the change in pre- to post-operative. We found that baseline values predicted the change in metric in 21/432 variables after correcting for Bonferroni correction.
